# Cationic Synthetic Peptides: Assessment of Their Antimicrobial Potency in Liquid Preserved Boar Semen

**DOI:** 10.1371/journal.pone.0105949

**Published:** 2014-08-22

**Authors:** Stephanie Speck, Alexandre Courtiol, Christof Junkes, Margitta Dathe, Karin Müller, Martin Schulze

**Affiliations:** 1 Leibniz Institute for Zoo and Wildlife Research, Berlin, Germany; 2 Leibniz Institute of Molecular Pharmacology, Berlin, Germany; 3 Institute for Reproduction of Farm Animals Schoenow e. V., Bernau, Germany; Institut National de la Recherche Agronomique, France

## Abstract

Various semen extender formulas are in use to maintain sperm longevity and quality whilst acting against bacterial contamination in liquid sperm preservation. Aminoglycosides are commonly supplemented to aid in the control of bacteria. As bacterial resistance is increasing worldwide, antimicrobial peptides (AMPs) received lively interest as alternatives to overcome multi-drug resistant bacteria. We investigated, whether synthetic cationic AMPs might be a suitable alternative for conventional antibiotics in liquid boar sperm preservation. The antibacterial activity of two cyclic AMPs (c-WWW, c-WFW) and a helical magainin II amide analog (MK5E) was studied *in vitro* against two Gram-positive and eleven Gram-negative bacteria. Isolates included ATCC reference strains, multi-resistant *E. coli* and bacteria cultured from boar semen. Using broth microdilution, minimum inhibitory concentrations were determined for all AMPs. All AMPs revealed activity towards the majority of bacteria but not against *Proteus* spp. (all AMPs) and *Staphylococcus aureus* ATCC 29213 (MK5E). We could also demonstrate that c-WWW and c-WFW were effective against bacterial growth in liquid preserved boar semen *in situ*, especially when combined with a small amount of gentamicin. Our results suggest that albeit not offering a complete alternative to traditional antibiotics, the use of AMPs offers a promising solution to decrease the use of conventional antibiotics and thereby limit the selection of multi-resistant strains.

## Introduction

Artificial insemination (AI) is the most commonly used assisted reproductive technology in swine industry [1]. For AI, short- or long-term semen extenders are used to process and store semen while maintaining sperm viability over days at 15 to 17°C. Bacteria are frequently found in freshly retrieved boar ejaculates but are detrimental to sperm quality and longevity particularly in liquid-preserved semen [2]–[4]. Up to 10^9^ colony forming units/mL ejaculate have been reported [5]–[7]. The most prevalent bacteria were Gram-negative with the majority belonging to the family *Enterobacteriaceae* [7], [8]. Bacterial contamination seems to have little effect on fecundity under natural mating conditions. However, processing and storage of extended semen for AI might facilitate bacterial growth and concentration-dependent spermicidal effects [9]. Besides a proper sanitation and hygiene management, antimicrobial substances, such as Aminoglycosides, are commonly supplemented to aid in the control of bacteria [7], [9], [10].

Bacteria are highly effective in adapting to changing environments [11] and due to an increasing spread of resistance to classic antibiotics there is a need for new antimicrobial alternatives [3], [12]. In recent studies, antimicrobial peptides (AMPs) have received considerable attention as candidates to overcome bacterial resistance [13]. AMPs are naturally occurring molecules with a broad spectrum of antimicrobial activity that rapidly kill their target cells [14]. Well-known AMPs are mammalian defensins, amphibian magainins, and insect cecropins but even bacteria and fungi produce cationic AMPs (lantibiotics, bacteriocins) [14]. Roughly 5,500 AMPs have been discovered, predicted or synthesized so far [15]. Fortunately, most cationic peptides do not induce resistant mutant strains *in vivo* [14]. Among the large variety of AMPs, short arginine (R)- and tryptophan (W)-rich cyclic peptides demonstrated high antimicrobial activity and low toxic effects against eukaryotic cells [16]. Furthermore, the interaction of these R- and W-rich cyclic hexapeptides with *E. coli* rapidly permeabilised the outer membrane of *E. coli* [16], [17].

The aim of our study was to evaluate whether selected synthetic AMPs are useful as substitutes for conventional antibiotics used in liquid boar sperm preservation. We describe the antimicrobial activity of two cationic cyclic peptides (c-WWW, c-WFW) [16] and a cationic helical magainin II amide analog (MK5E) [18] *in vitro* and in liquid preserved boar semen.

## Materials and Methods

### Synthetic cationic antimicrobial peptides

A helical magainin II amide derivative (MK5E) and two cyclic hexapeptides (c-WWW, c-WFW) were used in this study. The antimicrobial activity of these peptides against *E. coli* DH5α and *Bacillus subtilis* subsp. *spizizenii* DSM 347 (further referred to as *B. subtilis*) and their interaction with eukaryotic cells have been described in detail previously [16]–[18]. Peptides (Table 1) were obtained lyophilized from Biosyntan, Berlin, Germany. Stock solutions (400 µM) prepared in sterile distilled water were stored at –80°C until further use. The peptide synthesis was previously described in detail [16].

**Table 1:** Cationic synthetic peptides used in this study.

Abbreviation	Peptide sequence	MW (g/mol)
c-WFW	Cyclic (RRWFWR)	989.5
c-WWW	Cyclic (RRWWWR)	1027.2
MK5E	Ac-GIGKF IHAVK KWGKT FIGEI AKS-NH2	2515.1

alanine (A), arginine (R), glutamic acid (E), glycine (G), histidine (H), isoleucine (I), lysine (K), phenylalanine (F), serine (S), threonine (T), tryptophan (W), valine (V), MW – molecular weight. The linear peptide, MK5E is N-terminally acetylated (Ac) and C-terminally amidated (NH_2_).

### Antimicrobial susceptibility testing

#### *In vitro* antimicrobial activity of c-WFW, c-WWW, and MK5E

For the determination of *in vitro* Minimum Inhibitory Concentrations (MICs), broth microdilution was performed according to the Clinical and Laboratory Standards Institute (CLSI) standard M31-A3 [19] using cation-adjusted Mueller-Hinton-II-Bouillon (MHIIB; Merck, Darmstadt, Germany). All antimicrobial substances were tested in 96-well plates in triplicate. These experiments were independently repeated twice. Selected Gram-negative bacteria isolated from native boar semen in preceding studies (unpublished data) were used: *Enterobacter cloacae*, hemolytic *E. coli* (further referred to as *E. coli* HE), *Klebsiella* (*K.*) *pneumoniae*, *Proteus* (*P*.) *myxofaciens*, *P. vulgaris*. In addition, AMPs were tested on *E. coli* DH5α, *B. subtilis* DSM 347, and four gentamicin-resistant *E. coli* (kindly provided by Stefan Schwarz, FLI, Mariensee, Germany). All strains were grown on Columbia sheep-blood (5%) agar (CSBA; Oxoid, Wesel, Germany). Briefly, MHIIB containing 5×10^5^ CFU/mL was prepared for subsequent inoculation into 96-well plates containing the different peptide dilutions. The final peptide concentrations ranged from 100 µM−0.05 µM (1∶2 serial dilutions) as previously described [16]. Plates were sealed and incubated at 37°C for 18 to 24 h. The MIC of each tested AMP was defined as the lowest concentration exhibiting no visible growth compared to drug-free control wells. Turbidity was monitored with unaided eyes and a microplate reader at 600 nm. Gentamicin MICs were also determined (final concentration 0.113 µg/mL–116 µg/mL). As a quality control (QC) for broth microdilution, *E. coli* ATCC 25922 and *Staphylococcus* (*S*.) *aureus* ATCC 29213 were used as reference strains as recommended by CLSI [19]. Results were compared to the MIC QC ranges for broth microdilution (µg/mL) given by CLSI [19]. The test results were considered valid only when MICs for reference strains were within the QC ranges accepted by CLSI [19].

#### Evaluation of potency-enhancing effects: application of c-WWW and MK5E combined to gentamicin

The combination of AMPs and classical antibiotics has the potential to enhance the potency and target selectivity of AMPs [20]. We therefore combined c-WWW (2 µM) and MK5E (1 µM) but not c-WFW (as the latter was most promising for a stand-alone application) to gentamicin. AMP-concentrations were chosen according to sperm toxicity data as c-WWW and MK5E even at their lowest MIC (see results) would be harmful to boar spermatozoa (unpublished data). Gentamicin concentrations (i.e. 0.025 µg/mL–1 µg/mL) were selected according to MIC values defined in the first experiments and combined with c-WWW and MK5E. Determination of bacterial *in vitro* susceptibility was performed according to CLSI [19] and as outlined before. In addition, MICs were determined for gentamicin as a QC. The four multi-resistant *E. coli* were not included in these experiments.

### Detection of bacteria in preserved semen

Ejaculates were collected from mature Pietrain boars housed at an EU-approved commercial insemination center during routine semen production and not as an animal experiment. The approval number according to Directive 90/429/EEC is KBS 085-EWG. Samples originated from a total of 39 boars and were retrieved by the gloved-hand technique. The gelatinous ejaculate fraction was removed using gauze. Boar ejaculates were diluted in Beltsville Thawing Solution (BTS) without additives (Minitüb, Tiefenbach, Germany), split, adjusted to 2×10^9^ spermatozoa/portion (90 mL), and slowly cooled to 16°C over a 5 h-period.

The standard extender BTS containing 250 µg/mL gentamicin (BTS+G) was used as the control for all experiments. Ejaculates of ten individuals were comparatively investigated using BTS + c-WWW (2 µM) and BTS + c-WFW (4 µM). Samples of nine other individuals were prepared using BTS + MK5E (1 µM). In addition, a preparation using BTS without antimicrobial additive (BTS only) was available from three of these nine individuals. For the combined application of gentamicin (G) and AMPs, ejaculates from another 20 boars were prepared. BTS+G (16 µg/ml) was combined with c-WWW (2 µM), c-WFW (4 µM), and MK5E (1 µM), respectively. BTS+G (16 µg/mL) served as additional control. The latter concentration corresponded to the two-fold MIC breakpoint for gentamicin*-*resistant *Enterobacteriaceae* [19].

Each preparation was stored for 96 h at 16°C. Counting of bacteria and determination of bacterial species was performed after 12 h, 48 h, and 96 h of storage, respectively. To identify the different bacteria, a 50 µL-aliquot of the respective sample was each plated onto CSBA, Gassner medium (Oxoid), and McConkey agar (Oxoid). Plates were incubated for 48 h at 37°C. Bacterial species identification was carried out based on growth characteristics, Gram-staining, catalase- and oxidase-reaction, and conventional as well as commercially available (API^®^ test system, bioMérieux, Nürtingen, Germany) biochemical tests. In addition, a serial dilution (10^−1^ to 10^−5^) was prepared from each preparation after the respective storage time. 100 µL of each dilution were plated onto two nutrient agar plates (Oxoid), respectively. Plates were inspected after 24 h and 48 h of incubation at 37°C. Colony forming units (CFU)/mL were calculated after 48 h of incubation.

### Statistical analysis

To study the influence of AMPs on bacterial growth in preserved semen, we used the non-parametric test for longitudinal data in factorial experiments by Brunner *et al.* (2002) [21]. This test has specifically been designed to analyze time-dependent outcomes of an experiment performed on a small number of subjects. Analyses were implemented using the package nparLD version 2.1 [22] for the free statistical software R version 3.0.2 [23]. Following authors’ terminology, our experiment setting corresponded originally to a F0-LD-F2 design. This means that for each semen sample, that we consider as subjects, we have no between-subject covariate and two within-subject covariates: time and treatment. The response variable was the number of CFU/mL.

In order to compare the effect of BTS only, gentamicin and the three AMPs on bacterial growth, we pooled the ten ejaculates treated with BTS+G (250 µg/mL), BTS + c-WWW (2 µM) and BTS + c-WFW (4 µM) and the nine ejaculates treated with BTS+G (250 µg/mL), BTS + MK5E (1 µM) and BTS only (for three of those nine ejaculates) in a first analysis. As preserved semen from each animal was not treated by all five treatments, we could not run the analysis as a F0-LD-F2 design. Instead, we randomly selected one treatment for each animal, making sure that the random sampling always included at least one sample for each treatment, and considered treatment as a between-subject covariate (F1-LD-F1 design). When testing of the effect of treatments on bacterial growth, the outcome is subject to variation due to the random sampling procedure. Therefore, we replicated the analysis 1000 times and report the median of all 1000 p-values obtained (hereafter reported *simulated p-value*). Importantly, making a separate analysis for each experiment and respecting the initial F0-LD-F2 study design led to same qualitative conclusions but precludes one to compare all treatments together (analysis performed without the treatment BTS only as this latter was not applied on all ejaculates, data not shown).

We also reran this analysis excluding the preparation BTS+G (250 µg/mL) to study differences between AMPs. Then, we performed a second analysis for the 20 ejaculates treated with BTS+G (250 µg/mL), BTS+G (16 µg/mL), BTS+G (16 µg/mL) + c-WWW (2 µM), BTS+G (16 µg/mL) + c-WFW (4 µM) and BTS+G (16 µg/mL) + MK5E (1 µM) to study the effect of a combined application of gentamicin and AMPs. For this latter analysis, directly fitting a F0-LD-F2 model was possible because each subject received all treatments.

## Results

### Antimicrobial susceptibility testing using c-WWW, c-WFW, and MK5E

MICs (µg/mL) defined for gentamicin using *S. aureus* ATCC 29213 (i.e. 0.225–0.7 µg/mL) and *E. coli* ATCC 25922 (i.e. 0.45–0.9 µg/mL) were within QC range recommended by CLSI (*S. aureus* ATCC 29213 0.12-1 µg/mL, *E. coli* ATCC 25922 0.25-1 µg/mL) [19]. Test results were reproducible in all experiments. Hence, systematic errors could be excluded. MICs determined for AMPs are given in Table 2. For most bacteria, the lowest MICs were defined for c-WFW followed by c-WWW and the linear magainin derivative MK5E. Using *Proteus*, MIC values for all peptides exceeded 100 µM and were not further specified. *Enterobacter cloacae* revealed identical values for all three AMPs. MIC values determined for a certain bacteria/peptide combination did not differ within one experiment but small variation was observed between experiments. This has been expected as approved QC MIC values for standard antibiotics also span over a range of concentrations in broth microdilution [19].

**Table 2:** Minimum inhibitory concentrations (MICs) determined for synthetic cationic peptides.

	MICs (µM) determined for		
Bacteria	c-WFW	c-WWW	MK5E
*Escherichia coli* ATCC 25922	6.3–12.5	50	25–50
*Escherichia coli* DH5α	6.3	12.5–25	25–50
*Escherichia coli* (hemolytic)	6.3–12.5	50	25–50
*Escherichia coli* 26	12.5	25–50	50
*Escherichia coli* 629	6.3	25	25
*Escherichia coli* 2078	12.5	25	50
*Escherichia coli* 2715	12.5	25	25
*Enterobacter cloacae*	25	25	25
*Klebsiella pneumoniae*	12.5–25	25–50	50
*Proteus myxofaciens*	>100	>100	>100
*Proteus vulgaris*	>100	>100	>100
*Bacillus subtilis* DSM 347	6.3	6.3	6.3–12.5
*Staphylococcus aureus* ATCC 29213	25	50	>100

### Combination of c-WWW, MK5E and gentamicin

Addition of 2 µM c-WWW or 1 µM MK5E to varying concentrations of gentamicin resulted in MIC values that did not considerably differ from those obtained solely for gentamicin (Table 3). Compared to the latter, a slight increase of MICs was noticed with the exception of *B. subtilis* DSM 347 as test organism.

**Table 3:** Minimum inhibitory concentrations (MICs) determined for gentamicin when combined with c-WWW or MK5E.

	MIC (µg/mL) determined for gentamicin	MIC (µg/mL) determined for gentamicin when combined with	
Bacteria		c-WWW (2 µM)	MK5E (1 µM)
*Escherichia coli* ATCC 25922	0.45–0.9*	0.6–0.7	0.6–0.8
*Escherichia coli* DH5α	0.113	0.3–0.5	0.2–0.5
*Escherichia coli* (hemolytic)	0.45	0.8–0.9	0.9
*Enterobacter cloacae*	0.113–0.225	0.2–0.4	0.3–0.4
*Klebsiella pneumoniae*	0.225–0.45	0.4–0.7	0.5–0.6
*Proteus myxofaciens*	0.45–0.9	0.7–0.9	0.7–0.9
*Proteus vulgaris*	0.45	0.6–0.8	0.5–0.8
*Bacillus subtilis* DSM 347	0.113	0.05–0.1	0.1
*Staphylococcus aureus* ATCC 29213	0.225–0.7*	0.6–0.7	0.5–0.6

*QC ranges as recommended by CLSI [19]: *S. aureus* (ATCC 29213) 0.12–1 µg/mL and *E. coli* (ATCC 25922) 0.25–1 µg/mL.

### Effect of synthetic antimicrobial peptides on bacterial contamination in liquid preserved boar semen

**Figure 1:**
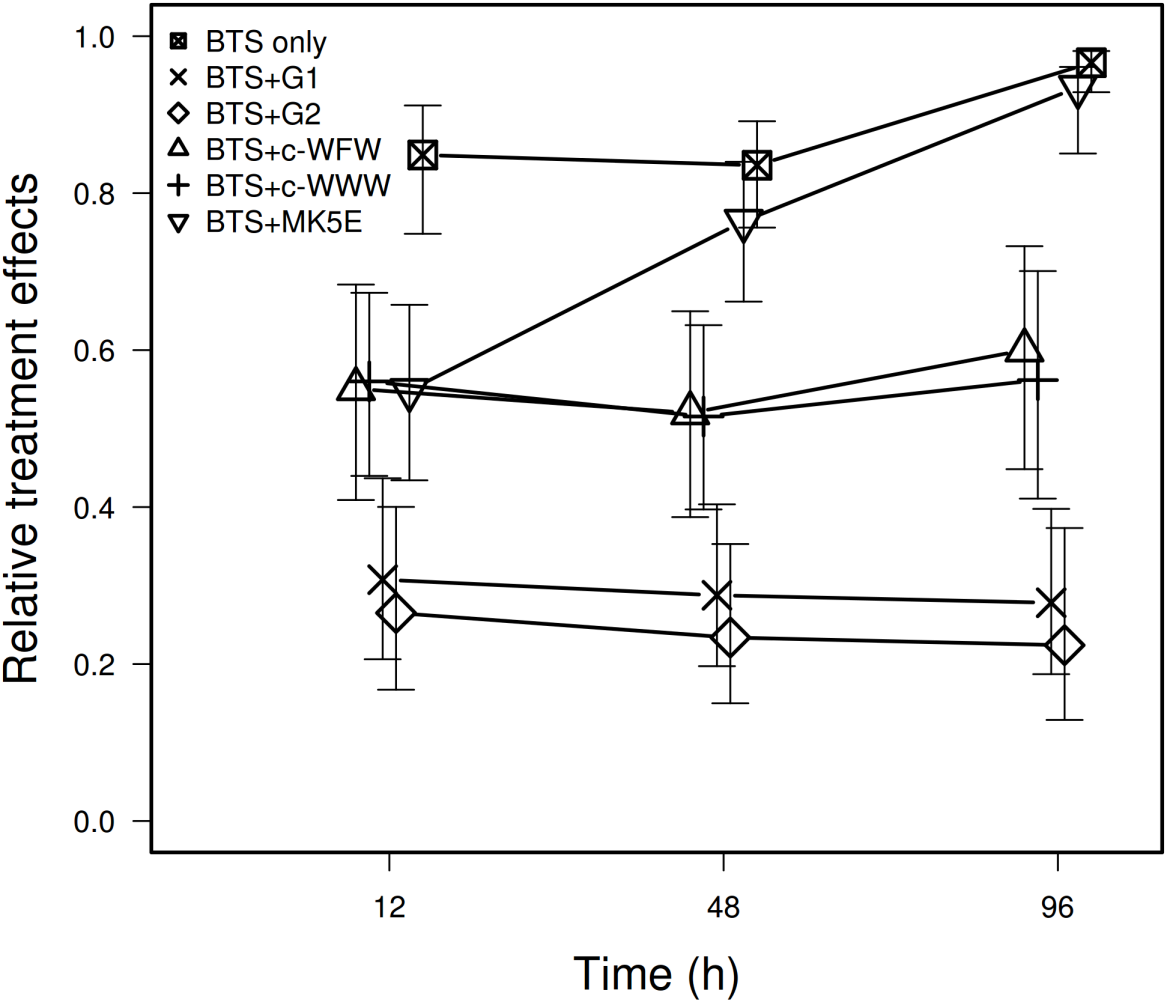
Relative effect of gentamicin or AMPs on the amount of bacteria in sperm preparation over time. Sperm preparations were made of BTS+G (250 µg/mL), BTS + c-WWW (2 µM) and BTS + c-WFW (4 µM) for ejaculates from ten individuals, and of BTS+G (250 µg/mL) and BTS + MK5E (1 µM) for ejaculates from nine other boars. Controls involving only BTS were also prepared from three of these nine individuals. The treatment BTS+G (250 µg/mL) is labeled BTS+G1 and BTS+G2 for the first and second experiment, accordingly. BTS+G1 and BTS + G2 were not distinguished in the analyses. The y-axis is the conventional graphical representation of the nonparametric method we used (see methods). It represents the relative marginal effect of the different treatments across time, i.e. the probability that the value being considered presents more CFU/mL than a random observation. The higher is the value on the y-axis, the higher is the corresponding value of CFU/mL, and the less effective is the treatment. Intervals represent 95% confidence intervals of the relative marginal effects and can here be used to compare treatments as the sample size is relatively similar for each point.

Ejaculates of ten boars prepared with BTS+G (250 µg/mL), BTS + c-WWW (2 µM) and BTS + c-WFW (4 µM) and of nine boars prepared with BTS+G (250 µg/mL) and BTS+MK5E (1 µM) were investigated. In addition, BTS only-preserved samples from three boars were studied. As shown in Figure 1, treatments with AMPs or gentamicin presented fewer bacteria than the BTS only control. The number of CFU/mL did not significantly change with time for any preparations but BTS only and MK5E (simulated p-value for Anova Type Statistic [ATS] of the effect of time: for BTS only p = 0.021; for MK5E p<0.001; for all other treatments: p>0.38). Meanwhile, there was significantly less CFU/mL observed when using BTS+G (i.e. the standard semen extender) compared to when using any of the three AMP preparations (Figure 1). The comparison of AMPs showed that all three preparations did not differ significantly when the entire length of the experiment is considered (p = 0.11), but as bacteria grew with time for the MK5E treatment, once the bacteria count at 12 h is discarded the difference between treatments becomes significant (simulated p-value for modified ATS of the effect of preparation: p = 0.015). At 48 h and 96 h, MK5E was a less effective treatment against bacteria than c-WWW and c-WFW (p<0.001) and lost the initial improvement it had over the BTS only control observed at 12 h. During the entire experiment, c-WWW and c-WFW did not differ between each other in CFU/mL observed (p = 0.8).

**Figure 2:**
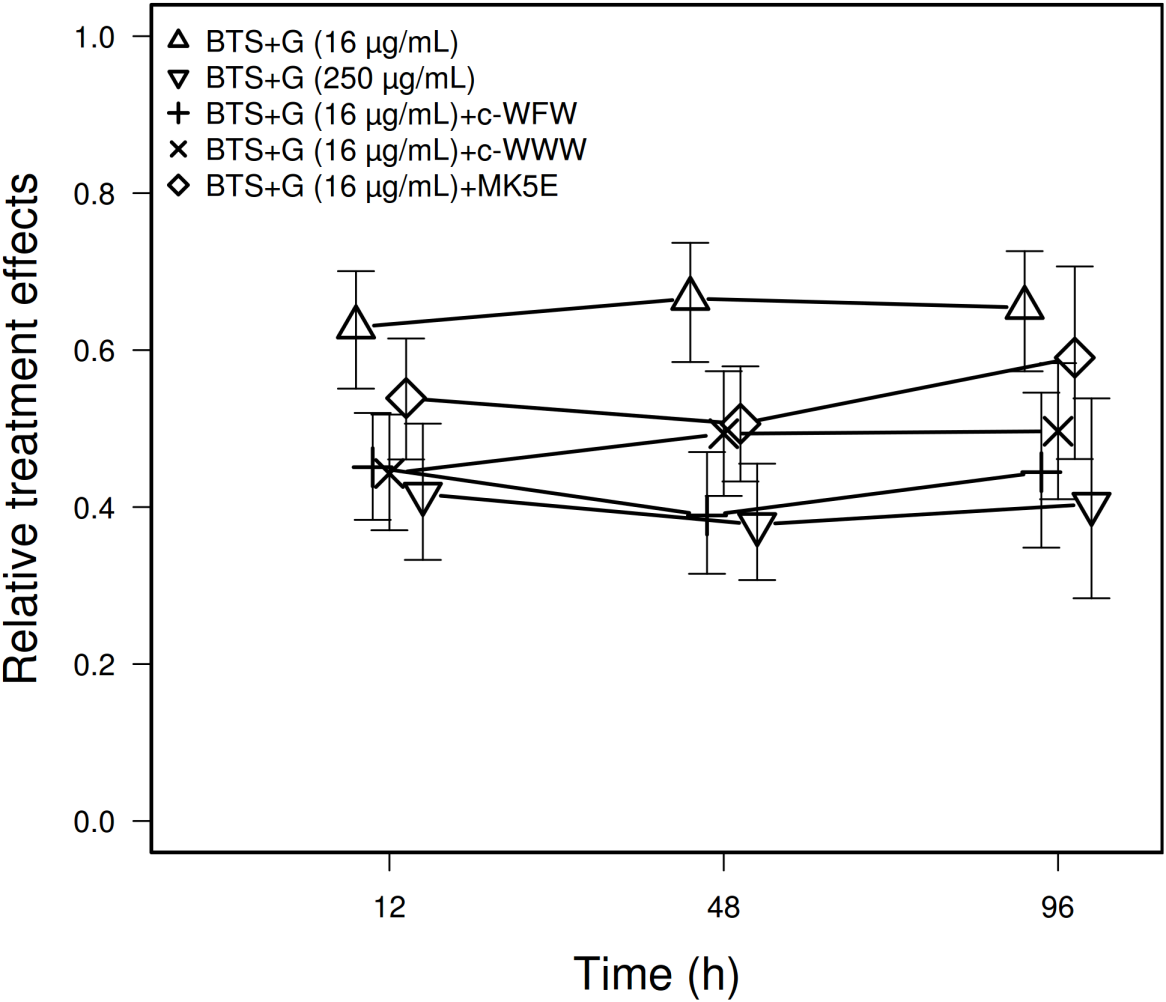
Relative effect of gentamicin or gentamicin combined with AMPs on the amount of bacteria in sperm preparation over time. Sperm preparations were made of BTS+G (250 µg/mL), BTS+G (16 µg/mL), BTS+G (16 µg/mL)+c-WWW (2 µM), BTS+G (16 µg/mL)+c-WFW (4 µM), and BTS+G (16 µg/mL)+MK5E (1 µM). See Figure 1 for legend details.

Ejaculates of 20 boars were prepared to evaluate the effects of BTS+G (16 µg/mL) + c-WWW (2 µM), BTS + G + c-WFW (4 µM), and BTS + G + MK5E (1 µM) compared to the standard BTS+G (250 µg/mL) and BTS+G (16 µg/mL). Figure 2 shows that the amount of CFU/mL did not seem to change with time for any of the combined AMP/gentamicin-preparations (ATS for main effect of time: 0.57, df = 1.58, p = 0.52; ATS for time interacting with treatment: 0.65, df = 4.22, p = 0.63). In contrast, the number of CFU/mL was influenced by the preparation (ATS: 9.51, df = 3.33, p<0.0001) with BTS+G (16 µg/mL) being the less effective treatment, followed by BTS+G (16 µg/mL)+MK5E. Best results were obtained from preparations containing BTS+G (16 µg/mL)+c-WFW, BTS+G (16 µg/mL)+c-WWW, and BTS+G (250 µg/mL). There was no significant difference in CFU/ml when using the latter three preparations (ATS: 1.63, df = 1.86, p = 0.20).

The amount of bacteria determined in different sperm preparations over time clearly varied between individuals. The CFU/mL counted for all preparations can be found in Table S1.

In total, 151 samples were investigated for bacterial growth. In the majority of samples (n = 125) more than one bacteria species was found. Scant growth of non-specific bacteria including mainly Gram-positive skin flora and Gram-negative bacteria commonly known as contaminants of distilled and stored water was found in 34% of all samples. Besides the non-specific bacteria, *Stenotrophomonas* (*S*.) *maltophilia* was predominant in samples treated solely by BTS+G (250 µg/mL, 16 µg/mL). Between three and five different Gram-negative and Gram-positive bacteria were isolated from the three BTS only preparations and identified as *S. maltophilia, Acinetobacter* sp., *Proteus* (*P*.) *vulgaris*, *Proteus* sp., *Serratia marcescens*, *Providencia rettgeri*, and *Staphylococcus* species. Preparations made of AMPs revealed ten different Gram-negative bacteria including *P. mirabilis*, *P*. *penneri*, *P*. *vulgaris, P. myxofaciens*, *Providencia alcalifaciens*, *Providencia rettgeri* or the non-fermentative bacteria *S. maltophilia*, *Ralstonia pickettii*, *Burkholderia cepacia*, and *Delftia acidovorans*. Of 29 samples treated solely with the single use of c-WWW, c-WFW, and MK5E, 21 (72%) revealed *Proteus* spp. and six (21%) were positive for *S. maltophilia*. In contrast, among the 60 samples obtained after the combined AMP/gentamicin treatment, we obtained eight (13%) *Proteus* spp.-positive specimens and 21 (35%) *S. maltophilia*-positive samples. Therefore, combining gentamicin (16 µg/mL) with an AMP significantly decreased the prevalence of *Proteus* spp. (proportion test: X^2^ = 28.4, df = 1, p<0.0001), but did not significantly influence *S. maltophilia* counts (X^2^ = 1.28, df = 1, p = 0.26).

## Discussion

Alternatives to conventional antibiotics are in urgent need to combat multidrug-resistant bacteria. Because of their effectiveness, antimicrobial peptides have been suggested for antimicrobial therapy [24]. The aim of our study was to investigate whether cationic AMPs are effective against bacteria often found in boar semen and therefore might be a suitable alternative to antibiotics currently used in liquid sperm preservation.

MICs could be determined *in vitro* for c-WFW, c-WWW, and MK5E using eleven bacterial strains with the exception of *Proteus* spp. (all AMPs) and *S*. *aureus* ATCC 29213 (MK5E). These latter bacteria are known to produce proteases that cleave naturally occurring linear cationic AMPs [24] and this mechanism might contribute to the results obtained in our experiments. Of the three peptides investigated, c-WFW resulted in lowest MIC values followed by c-WWW and MK5E. In former studies, hemolytic activity as well as toxicity against human cells at peptide concentrations up to 200 µM was negligible (c-WWW, c-WFW) [16] to non-existent (MK5E) [18]. However, our MIC-results revealed that only c-WFW might be applicable in liquid sperm preservation as negative effects on boar spermatozoa appeared at peptide concentrations higher than the MIC determined in this study (unpublished data). In contrast, even the lowest c-WWW and MK5E MIC determined for Gram-negative and -positive bacteria would be harmful to boar spermatozoa (unpublished data). We further investigated, whether a combined application of gentamicin and AMPs would result in enhanced antimicrobial effectiveness. For these experiments sperm-compatible concentrations of c-WWW and MK5E but not c-WFW (as the latter was most promising for a stand-alone application) were used. Results of the combined application revealed bactericidal activity when c-WWW (2 µM) and MK5E (1 µM) were combined with gentamicin at a concentration of <1 µg/mL. However, MIC values defined for gentamicin in the combined application were slightly higher than those obtained solely for gentamicin. Hence, we cannot deduce an enhancing effect from the results of these experiments *in vitro*. In fact, the increase of gentamicin MICs in the presence of AMPs would rather indicate an antagonistic effect. Cell membrane interaction is the first and most crucial step for the antimicrobial activity of AMPs [17]. Cationic charge and amphipathicity of AMPs constitute the structural prerequisite for an initial electrostatic interaction with negatively charged lipid systems [25]. Electrostatic interactions are also the first step in aminoglycoside (e.g. gentamicin) action [26], hence a competing effect between both molecules might be assumed resulting in apparently higher MICs *in vitro*.

Based on the fact that, with the exception of c-WFW, MIC values determined for c-WWW and MK5E would be detrimental to spermatozoa, we decided to use sperm-compatible AMP concentrations to investigate whether their use in liquid sperm preservation would have any effect on bacterial contamination *in situ*. Treatments with AMPs or gentamicin presented fewer bacteria than the BTS only control. Interestingly, although used at concentrations below MIC determined *in vitro,* the different AMPs influenced the number of CFU/mL in liquid-preserved semen *in situ*. CFU/mL in preparations made of standard extender BTS containing 250 µg/mL gentamicin did not seem to change over time as was also the case for c-WFW and c-WWW that presented both the same antibacterial power (Figure 1). In contrast, preparations containing MK5E (1 µM) were less efficient and no longer prevented bacteria growth after 12 h. Enhancement of AMP-potency and target selectivity when combined to conventional antibiotics has been described [20] and might be affirmed by our data regarding AMP/gentamicin-preserved sperm *in situ* although this is not supported by our *in vitro* data. Figure 2 clearly demonstrates that the combination of gentamicin and c-WFW as well as c-WWW enhanced the antimicrobial effectiveness *in situ*. In fact, the standard BTS+G (250 µg/mL) was as effective as preparations made of gentamicin (16 µg/mL) + c-WFW as well as gentamicin (16 µg/mL) + c-WWW. This effect cannot be attributed to gentamicin alone because BTS containing 16 µg/mL gentamicin without AMPs was significantly less effective than all other preparations in this study. Therefore, our results suggest that albeit not offering a complete alternative to traditional antibiotics, the use of adequate AMPs may allow for a substantial reduction in concentration of antibiotics used for semen preservation.

The bacteria isolated from liquid extended boar semen confirmed findings reported by others [7], [9], [27]. In their studies, also *Enterobacteriaceae*, *Xanthomonadaceae*, *Alcaligenaceae*, and *Burkholderiaceae* accounted for most of the Gram-negative contaminants. Most of the bacteria we isolated originate from the boar or occur ubiquitously and are often associated with water. Many of them have an inherent ability to form biofilms and possess intrinsic or acquired resistance mechanisms. Overall, approximately one third of all samples contained *Enterobacteriaceae or S. maltophilia*. Althouse *et al.* [9] stated that ejaculates contaminated by bacteria only have little effect on fecundity under natural mating conditions. However, the presence of *S. maltophilia* was directly correlated to sperm agglutination and decreased gross motility [9]. Other Gram-negative bacteria may also act spermicidal thus negatively affecting litter size, when sows are inseminated with contaminated semen [27].

The usage of AMPs in liquid semen preservation was hindered by their sperm-toxicity at higher concentrations (unpublished data). Unexpectedly, we found AMPs effective *in situ* at concentrations that deemed to be ineffective during screening *in vitro*. We chose performance standards for antimicrobial dilution susceptibility tests according to CLSI [19] for quality assurance. Cation-adjusted MHIIB is recommended when using gentamicin as a reference [19] but may affect AMP properties. Cation-adjusted MHIIB contains 20 to 25 mg/L Ca^2+^ and 10 to 12.5 mg/L Mg^2+^ who might influence AMP-target structure-interactions. With regard to the magainin II amide analog MK5E this is supported by results of Matsuzaki *et al.* (1999) [28] who reported that Mg^2+^ tightens the lipopolysaccharide (LPS) packing by crosslinking adjacent phosphate groups. Their studies showed that 10 mM Mg^2+^ blocked the bactericidal action of magainin 2 on membrane models *in vitro* [28]. The *in situ* effect seen in our study might be explained by the finding that the antimicrobial activity of AMPs depends on an ionic milieu comparable to that in mammalian body fluids [29]. This was demonstrated on a structurally diverse panel of AMPs [29]. The presence of NaHCO_3_ (27 mM) significantly enhanced antimicrobial activity against Gram-positive and -negative bacteria [29]. It has also been suggested that carbonate enhances AMP activity due to alterations in bacterial susceptibility [29]. Besides other components to preserve sperm metabolic activity (e.g. 3.7 mM EDTA), the standard extender BTS we used contained 15 mM NaHCO_3_ thus possibly enhancing microbial susceptibility to AMPs in liquid-preserved semen.

## Conclusions

Our results demonstrate activity of synthetic cationic antimicrobial peptides against different Gram-negative and Gram-positive bacteria *in vitro*. Furthermore, c-WWW and c-WFW suppressed bacterial growth in semen preparations *in situ*, especially when combined with a small concentration of gentamicin. As we also examined that AMPs did not impede the quality of sperm (unpublished data), they offer a promising solution to decrease the use of conventional antibiotics and thereby limit the selection of multi-resistant strains. In order to achieve comparable data for *in vitro* susceptibility testing and *in situ* studies, the implementation of a valid standardized method is in need. With regard to the application of AMPs in liquid boar sperm preservation further investigations should include the reduction of sperm toxicity, detection of possible enhancing effects using other conventional antibiotics, and analyses of peptide-stability in different standard semen extenders.

## Supporting Information

Table S1 **Bacterial counts given in CFU/mL in different sperm preparations determined after 12 h, 48 h, and 96 h of storage at 16°C.**
(DOCX)
