# Supplementary material for: Cationic Synthetic Peptides: Assessment of Their Antimicrobial Potency in Liquid Preserved Boar Semen
Source: PLoS One. 2014 Aug 22;9(8):e105949. doi: 10.1371/journal.pone.0105949 (PMC4141845; doi:10.1371/journal.pone.0105949)
Supplement: Table S1 — Bacterial counts given in CFU/mL in different sperm preparations determined after 12 h, 48 h, and 96 h of storage at 16°C. (DOCX) [file pone.0105949.s001.docx]

**Table S1. Bacterial counts (CFU/mL) in different sperm preparations after 12 h, 48 h, and 96 h of storage at 16 °C.**

| **Animal** | **BTS+G (250 µg/mL)** | | | **BTS+c-WWW (2 µM)** | | | **BTS+c-WFW (4 µM)** | | |
| --- | --- | --- | --- | --- | --- | --- | --- | --- | --- |
|  | **CFU/mL** | | | **CFU/mL** | | | **CFU/mL** | | |
|  |  |  |  |  |  |  |  |  |  |
|  | **12 h** | **48 h** | **96 h** | **12 h** | **48 h** | **96 h** | **12 h** | **48 h** | **96 h** |
|  |  |  |  |  |  |  |  |  |  |
| A | 40 | 300 | 150 | 414 | 180 | 85 | 264 | 100 | 173 |
| B | 285 | 145 | 209 | 325 | 210 | 4591 | 277 | 220 | 973 |
| C | 40 | 30 | 0 | 3350 | 3300 | 2762 | 3400 | 3300 | 2800 |
| D | 0 | 0 | 0 | 35 | 100 | 20 | 30 | 1 | 30 |
| E | 20 | 1 | 40 | 20 | 10 | 30 | 40 | 20 | 30 |
| F | 1 | 20 | 70 | 170 | 168 | 6300 | 1 | 323 | 8200 |
| G | 29 | 35 | 0 | 319 | 55 | 630 | 450 | 105 | 3100 |
| H | 52 | 35 | 20 | 339 | 65 | 140 | 550 | 143 | 275 |
| I | 35 | 20 | 59 | 76 | 500 | 64 | 281 | 978 | 45 |
| V | 640 | 165 | 75 | 952 | 809 | 300 | 1200 | 673 | 722 |
|  |  |  |  |  |  |  |  |  |  |
| **Animal** | **BTS only** | | | **BTS+G (250 µg/mL)** | | | **BTS+MK5E (2 µM)** | | |
|  | **CFU/mL** | | | **CFU/mL** | | | **CFU/mL** | | |
|  |  |  |  |  |  |  |  |  |  |
|  | **12 h** | **48 h** | **96 h** | **12 h** | **48 h** | **96 h** | **12 h** | **48 h** | **96 h** |
|  |  |  |  |  |  |  |  |  |  |
| J | na | na | na | 46 | 20 | 0 | 180 | 196 | 16400 |
| K | na | na | na | 0 | 0 | 0 | 109 | 1524 | 215000 |
| L | na | na | na | 0 | 36 | 20 | 124 | 208 | 10000 |
| M | na | na | na | 48 | 60 | 62 | 77 | 25454 | 360000 |
| N | na | na | na | 25 | 20 | 0 | 136 | 5667 | 6,1x10^6^ |
| O | na | na | na | 0 | 0 | 20 | 91 | 1937 | 1x10^8^ |
| P | 959 | 1762 | 50000 | 29 | 0 | 0 | 427 | 746 | 6600 |
| Q | 3364 | 5227 | 50000 | 200 | 76 | 205 | 1200 | 3200 | 92000 |
| R | 6182 | 1380 | 9,4x10^6^ | 299 | 261 | 232 | 886 | 882 | 776 |

na – sample not available

| **Animal** | **BTS+G (250 µg/mL)** | | | **BTS+G  (16 µg/mL)** | | | **BTS+G (16 µg/mL) +c-WFW (4 µM)** | | | **BTS+G (16 µg/mL) +c-WWW (2 µM)** | | | **BTS+G (16 µg/mL) +MK5E (1 µM)** | | |
| --- | --- | --- | --- | --- | --- | --- | --- | --- | --- | --- | --- | --- | --- | --- | --- |
|  | **CFU/mL** | | | **CFU/mL** | | | **CFU/mL** | | | **CFU/mL** | | | **CFU/mL** | | |
|  |  |  |  |  |  |  |  |  |  |  |  |  |  |  |  |
|  | **12 h** | **48 h** | **96 h** | **12 h** | **48 h** | **96 h** | **12 h** | **48 h** | **96 h** | **12 h** | **48 h** | **96 h** | **12 h** | **48 h** | **96 h** |
|  |  |  |  |  |  |  |  |  |  |  |  |  |  |  |  |
| 1 | 580 | 0 | 0 | 84 | 820 | 115 | 20 | 0 | 0 | 57 | 233 | 30 | 60 | 73 | 2x10^6^ |
| 2 | 0 | 0 | 0 | 40 | 25 | 20 | 0 | 0 | 30 | 57 | 40 | 0 | 0 | 36 | 20 |
| 3 | 0 | 0 | 0 | 52 | 43 | 42 | 0 | 0 | 20 | 30 | 65 | 30 | 223 | 20 | 25 |
| 4 | 100 | 55 | 55 | 95 | 176 | 91 | 55 | 35 | 30 | 45 | 35 | 20 | 71 | 90 | 140 |
| 5 | 57 | 41 | 25 | 177 | 344 | 146 | 82 | 60 | 40 | 25 | 129 | 67 | 35 | 50 | 20 |
| 6 | 40 | 52 | 0 | 60 | 40 | 20 | 20 | 45 | 20 | 30 | 62 | 30 | 35 | 50 | 0 |
| 7 | 25 | 30 | 20 | 238 | 131 | 143 | 218 | 150 | 162 | 33 | 76 | 40 | 200 | 195 | 127 |
| 8 | 67 | 60 | 30 | 110 | 272 | 255 | 91 | 191 | 1700 | 55 | 67 | 72000 | 259 | 29 | 24000 |
| 9 | 55 | 281 | 3900 | 80 | 133 | 91 | 100 | 155 | 138 | 20 | 123 | 90 | 95 | 105 | 30 |
| 10 | 40 | 20 | 40 | 138 | 219 | 205 | 50 | 45 | 20 | 30 | 28 | 55 | 65 | 100 | 91 |
| 11 | 85 | 81 | 65 | 405 | 396 | 314 | 227 | 11 | 30 | 236 | 300 | 346 | 136 | 114 | 110 |
| 12 | 150 | 668 | 5000 | 155 | 168 | 100 | 195 | 124 | 136 | 205 | 209 | 300 | 141 | 114 | 138 |
| 13 | 125 | 67 | 164 | 455 | 736 | 1100 | 105 | 113 | 605 | 182 | 232 | 2000 | 446 | 1300 | 26000 |
| 14 | 132 | 434 | 2500 | 671 | 566 | 550 | 159 | 236 | 127 | 300 | 296 | 930 | 406 | 1600 | 490000 |
| 15 | 118 | 434 | 2500 | 432 | 714 | 655 | 155 | 118 | 264 | 282 | 480 | 3200 | 486 | 2900 | 31000 |
| 16 | 1x10^6^ | 30 | 1,6x10^6^ | 30 | 1,5x10^8^ | 3,9x10^8^ | 20 | 0 | 20 | 30 | 0 | 0 | 0 | 0 | 748 |
| 17 | 20 | 20 | 0 | 213 | 55 | 295 | 236 | 96 | 43 | 114 | 30 | 43 | 75 | 65 | 0 |
| 18 | 30 | 0 | 0 | 125 | 138 | 100 | 25 | 0 | 20 | 45 | 25 | 0 | 100 | 81 | 75 |
| 19 | 0 | 0 | 0 | 96 | 25 | 105 | 0 | 20 | 0 | 0 | 0 | 60 | 30 | 0 | 26000 |
| 20 | 0 | 0 | 0 | 35 | 25 | 55 | 36 | 0 | 305 | 20 | 0 | 65 | 20 | 30 | 30 |
